# Robot-Assisted Resection of a Superior Prevascular Mediastinal Ectopic Parathyroid Adenoma by Subxiphoid Approach: A Case Report

**DOI:** 10.70352/scrj.cr.26-0121

**Published:** 2026-04-22

**Authors:** Saki Tsubouchi, Shohei Mori, Satoshi Arakawa, Naoki Toya, Takashi Ohtsuka

**Affiliations:** 1 Department of Surgery, The Jikei University Kashiwa Hospital, Kashiwa, Chiba, Japan; 2 Department of Surgery, Division of Thoracic Surgery, The Jikei University School of Medicine, Tokyo, Japan

**Keywords:** ectopic parathyroid adenoma, mediastinum, robot-assisted surgery, subxiphoid approach, radio-navigation

## Abstract

**INTRODUCTION:**

Ectopic mediastinal parathyroid adenoma is a rare cause of primary hyperparathyroidism, which is characterized by the excessive secretion of parathyroid hormone (PTH). Complete surgical resection is essential for curative treatment, making accurate intraoperative localization of the tumor a critical challenge. While superior mediastinal tumors have traditionally required invasive procedures such as median sternotomy, minimally invasive techniques, including video-assisted thoracoscopic surgery and robot-assisted surgery, have recently been adopted. However, in the lateral approach, visualization and instrument maneuverability beyond the left brachiocephalic vein are limited. In this case, we employed a robot-assisted subxiphoid approach combined with radio-navigation and intraoperative frozen section diagnosis for a superior prevascular mediastinal tumor adjacent to the jugular notch.

**CASE PRESENTATION:**

An 82-year-old male was referred with primary hyperparathyroidism. Imaging, including ^99m^Tc-methoxyisobutylisonitrile scintigraphy and contrast-enhanced CT, identified a 10-mm nodule in the superior prevascular mediastinum adjacent to the jugular notch. We performed a robot-assisted resection by subxiphoid approach using radio-navigation with a gamma probe and intraoperative frozen section diagnosis. The gamma probe was used to confirm the presence of target tissue in the excised specimen, and frozen section diagnosis confirmed that it was parathyroid tissue. The patient’s intact PTH levels normalized immediately postoperatively.

**CONCLUSIONS:**

The robot-assisted subxiphoid approach was feasible and provided an excellent surgical field for the superior prevascular mediastinum in this case. The combination of intraoperative radio-navigation and frozen section diagnosis is highly effective for ensuring the definitive resection of ectopic parathyroid adenomas.

## INTRODUCTION

Ectopic mediastinal parathyroid adenomas overproduce parathyroid hormone (PTH), leading to primary hyperparathyroidism.^1,2)^ While ectopic cases occur in approximately 20% of patients with parathyroid adenomas, those located in the mediastinum are relatively rare, accounting for only 1%–2% of cases.^3)^ Historically, these tumors required a sternotomy for resection; however, minimally invasive techniques such as thoracoscopic or robotic surgery have recently become more common. However, in the lateral approach, visualization and instrument maneuverability beyond the left brachiocephalic vein, particularly near the jugular notch, are limited. We report a case of a superior prevascular mediastinal ectopic parathyroid adenoma successfully resected via a robot-assisted subxiphoid approach combined with radio-navigation.

## CASE PRESENTATION

**Fig. 1:**
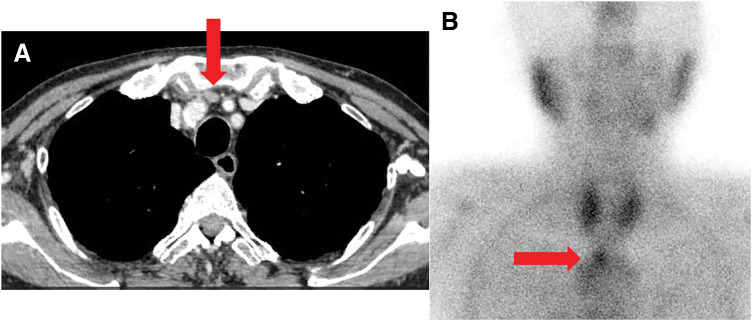
(**A**) Contrast-enhanced chest CT scan (axial view). A 10-mm well-defined nodule (red arrow) is identified in the superior mediastinum. (**B**) ^99m^Tc-MIBI scintigraphy. The delayed image reveals a significant nodular accumulation (red arrow) in the superior mediastinal region, consistent with an ectopic parathyroid adenoma. ^99m^Tc-MIBI, ^99m^Tc-methoxyisobutylisonitrile

An 82-year-old male presented with primary hyperparathyroidism discovered during an evaluation for ulcerative colitis. Although PTH measurement is not routine for ulcerative colitis, the detection of hypercalcemia led to further investigation, which confirmed the diagnosis of primary hyperparathyroidism with an elevated intact PTH level. Laboratory tests revealed elevated intact PTH 196 pg/mL (reference: 10–65 pg/mL), serum calcium 12.1 mg/dL (8.5–10.2 mg/dL), and decreased phosphorus 2.0 mg/dl (2.7–4.6 mg/dL). Renal dysfunction was noted with a creatinine level of 2.15 mg/dL. Contrast-enhanced CT showed a 10-mm nodule ventral to the trachea, located between the brachiocephalic artery and the left common carotid artery (**Fig. 1A** and **Supplementary Video 1**). ^99m^Tc-methoxyisobutylisonitrile (^99m^Tc-MIBI) scintigraphy showed a nodular accumulation caudal to the right thyroid lobe in the superior mediastinum (**Fig. 1B**).

Surgical procedure (**Supplementary Video 2**): The patient underwent robot-assisted resection via a subxiphoid approach. Under general anesthesia, the patient was placed in a supine position. Using the da Vinci Xi system (Intuitive Surgical Inc., Sunnyvale, CA, USA), we utilized three 8-mm intercostal ports (right 6th anterior axillary line; left 6th midclavicular and midaxillary lines), and one port of subxiphoid. Following CO_2_ insufflation maintained at 8–10 mmHg, retrosternal dissection was performed. To optimize the surgical field, the internal thoracic vein was divided, and the mediastinal pleura was incised from the suprasternal notch to the subxiphoid area.

Intraoperatively, we used a gamma probe (radio-navigation) to examine the resected specimen. An initial specimen showed only 25 counts per second (cps) and was confirmed as fat by frozen section analysis. Upon further exploration, a brownish nodule was identified more cranially than preoperative assessments had suggested. This nodule showed 371 cps on the gamma probe, and frozen section analysis confirmed it was parathyroid tissue. Total operative time was 276 min with minimal blood loss.

**Fig. 2:**
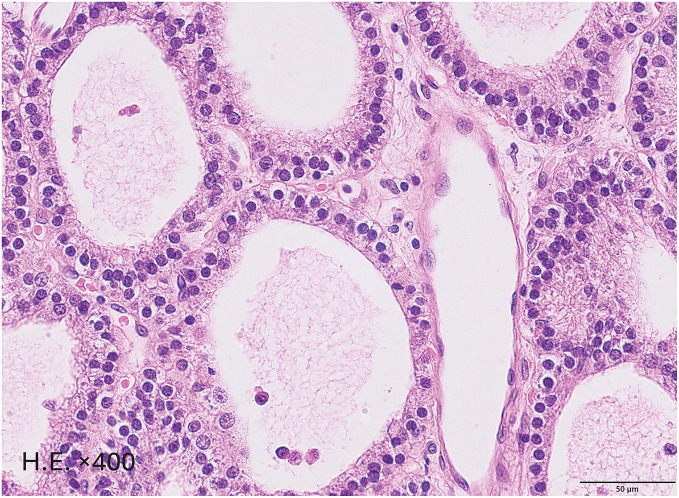
Histopathological findings (H.E. stain). It shows a nodule encapsulated by a thin fibrous capsule and the proliferation of atypical cells resembling chief cells, arranged in a follicular pattern with a rich capillary network. These findings are characteristic of a parathyroid adenoma. H.E., hematoxylin and eosin

Pathological findings: The specimen was a 10 × 8 × 5-mm yellowish-white nodule. Histopathological examination showed follicular growth of atypical chief-cell–like cells with a rich capillary network, consistent with a parathyroid adenoma (**Fig 2**). There were no findings of malignancy.

Postoperative course: Immediately after surgery, intact PTH normalized to 6 pg/mL. The chest tube was removed on the first POD. The patient was discharged home on POD 25 after management for a flare-up of ulcerative colitis. At the 5-month follow-up, calcium and intact PTH levels remained within normal ranges (9.0 mg/dL and 36 pg/mL, respectively).

## DISCUSSION

In this case, we successfully resected the superior prevascular mediastinal ectopic parathyroid adenoma using a robot-assisted subxiphoid approach. Although the operative time (276 min) was relatively long, this was primarily due to the challenges of intraoperative localization. Specifically, a second resection was required after the first specimen was pathologically confirmed as fat, demonstrating that intraoperative pathology remains essential for definitive resection despite the excellent visualization provided by the subxiphoid approach. Radio-navigation and frozen section diagnosis were also useful for ensuring the accuracy of the resection.

For symptomatic patients with excessive PTH secretion, minimally invasive surgical resection of ectopic mediastinal parathyroid adenoma is recommended as the standard of care by both the American Association of Endocrine Surgeons (AAES) and the National Institute for Health and Care Excellence (NICE).^4,5)^ Historically, surgical access to adenomas located in the superior prevascular mediastinum necessitated invasive procedures such as median sternotomy or manubriotomy. Recently, video-assisted thoracoscopic surgery (VATS) and robot-assisted thoracic surgery (RATS) have emerged as less invasive alternatives. In this case, we successfully employed a robot-assisted subxiphoid approach. Compared to the lateral intercostal approach, the subxiphoid approach provides a symmetrical, midline perspective of the superior mediastinal structures, including the bilateral recurrent laryngeal nerves, great vessels, and the trachea. This orientation is particularly superior for visualizing structures near the jugular notch and cranial to the left brachiocephalic vein, allowing for safer dissection around the great vessels compared to conventional lateral thoracoscopy. Utilizing the magnified 3D view and the articulated instrumentation of the da Vinci Xi system, we were able to maintain excellent visibility and maneuverability even in the narrow space cranial to the left brachiocephalic vein. While several reports have discussed robotic mediastinal parathyroidectomy,^6,7)^ to our knowledge, this is the first report describing a robot-assisted subxiphoid approach specifically for an ectopic adenoma in the superior mediastinum within the prevascular compartment, adjacent to the jugular notch.

Furthermore, we demonstrated that the integration of intraoperative radio-navigation (gamma probe guidance) and frozen section diagnosis is extremely effective for accurate tissue removal. Various methods for intraoperative identification have been reported, including intraoperative PTH monitoring (ioPTH), methylene blue staining, and near-infrared fluorescence imaging.^8–15)^ Radio-navigation using ^99m^Tc-MIBI provides immediate, quantitative real-time feedback. In this case, we utilized the gamma probe primarily for ex vivo specimen confirmation rather than intrathoracic navigation to avoid false-positive signals from the heart and great vessels. This quantitative feedback (371 cps), combined with frozen section analysis, ensured the complete resection of the true adenoma located at the suprasternal notch.

## CONCLUSIONS

The robot-assisted subxiphoid approach for an ectopic superior mediastinal parathyroid adenoma was feasible and provided an excellent surgical field in this case. The integration of radio-navigation and intraoperative pathology is highly effective for ensuring complete tumor resection.

## SUPPLEMENTARY MATERIALS

Supplementary Video 1 Contrast-enhanced chest CT scan. Axial view and 3D-CT reconstruction.

Supplementary Video 2 Intraoperative robotic procedure and radio-navigation.

## DECLARATIONS

### Availability of data and materials

Data sharing is not applicable to this article as no datasets were generated or analyzed during the current study.
